# Supplementary figures and images for: Separation of Donor and Recipient Microbial Diversity Allows Determination of Taxonomic and Functional Features of Gut Microbiota Restructuring following Fecal Transplantation
Source: mSystems. 2021 Aug 17;6(4):e00811-21. doi: 10.1128/mSystems.00811-21 (PMC8407411; doi:10.1128/mSystems.00811-21)

# A

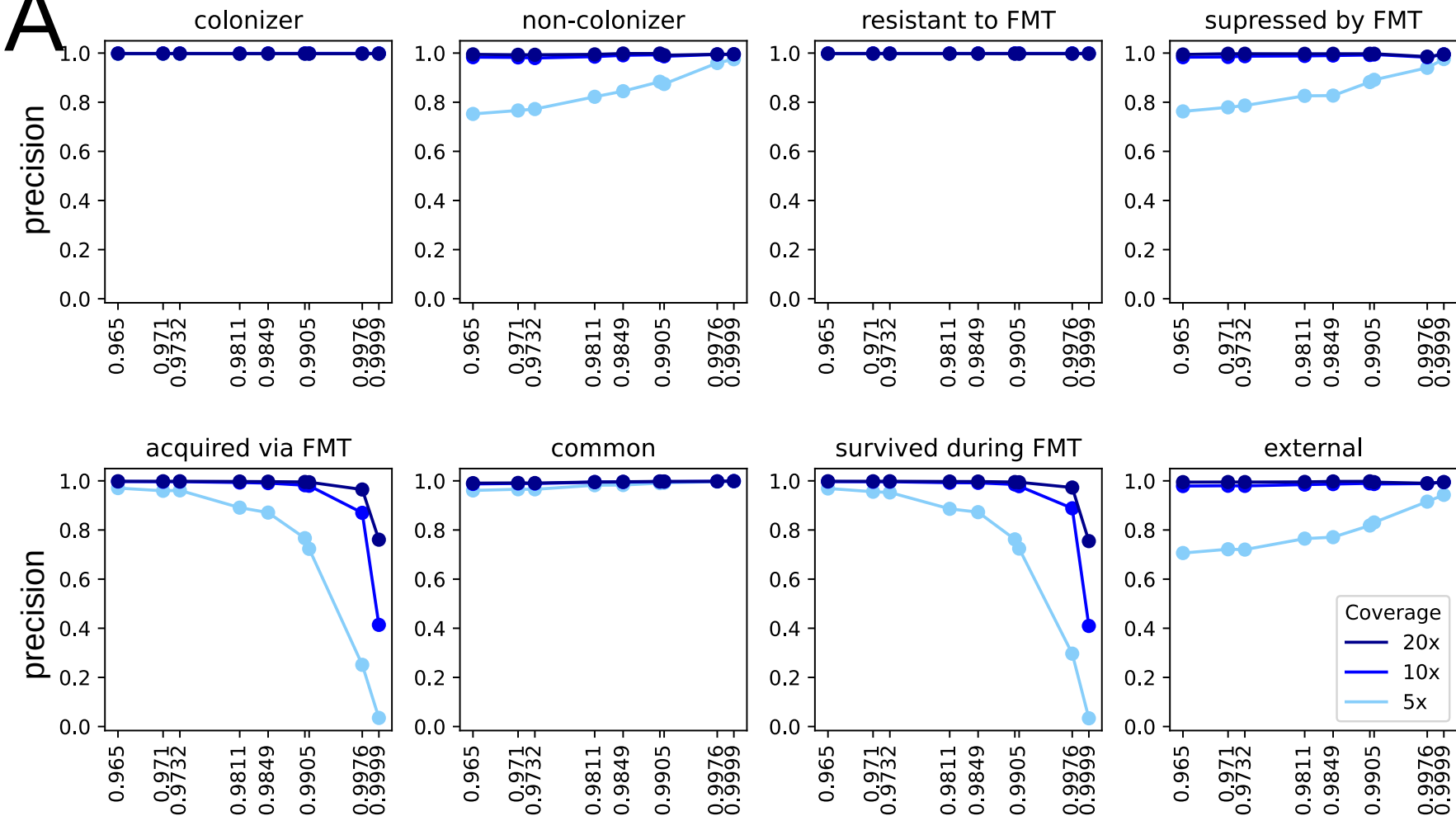

# B

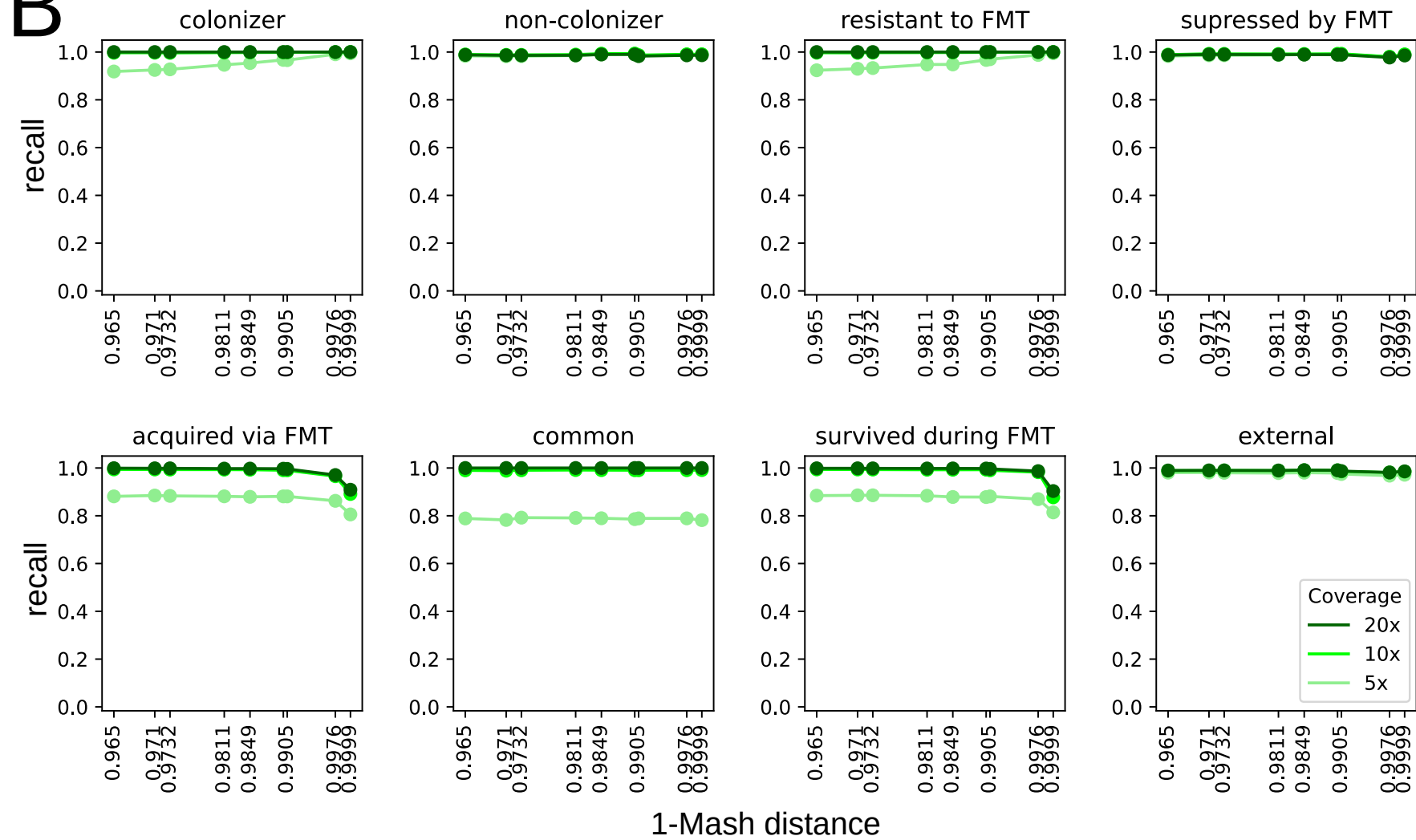

Supplement: FIG S1 [file msystems.00811-21-sf001.pdf]

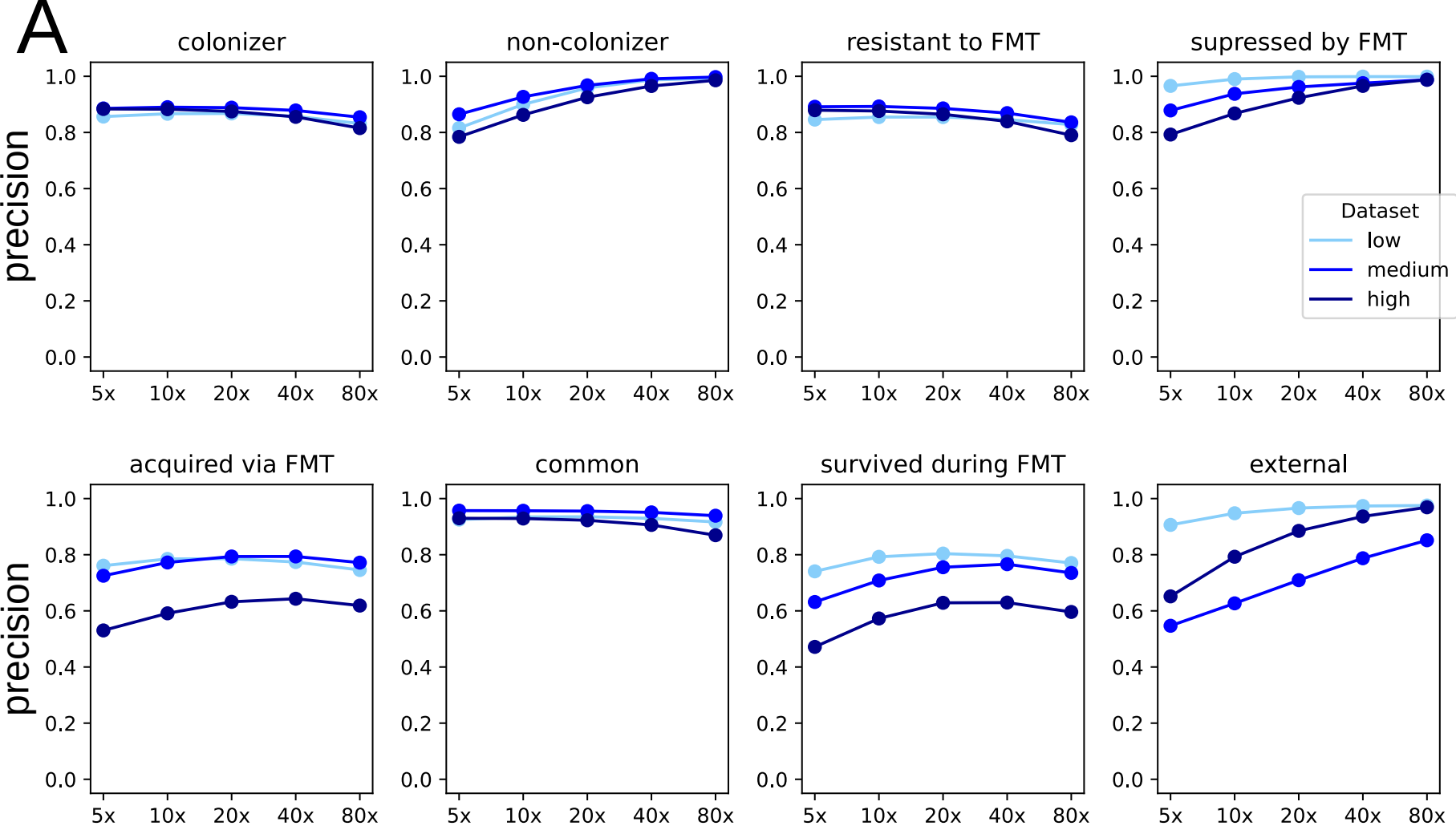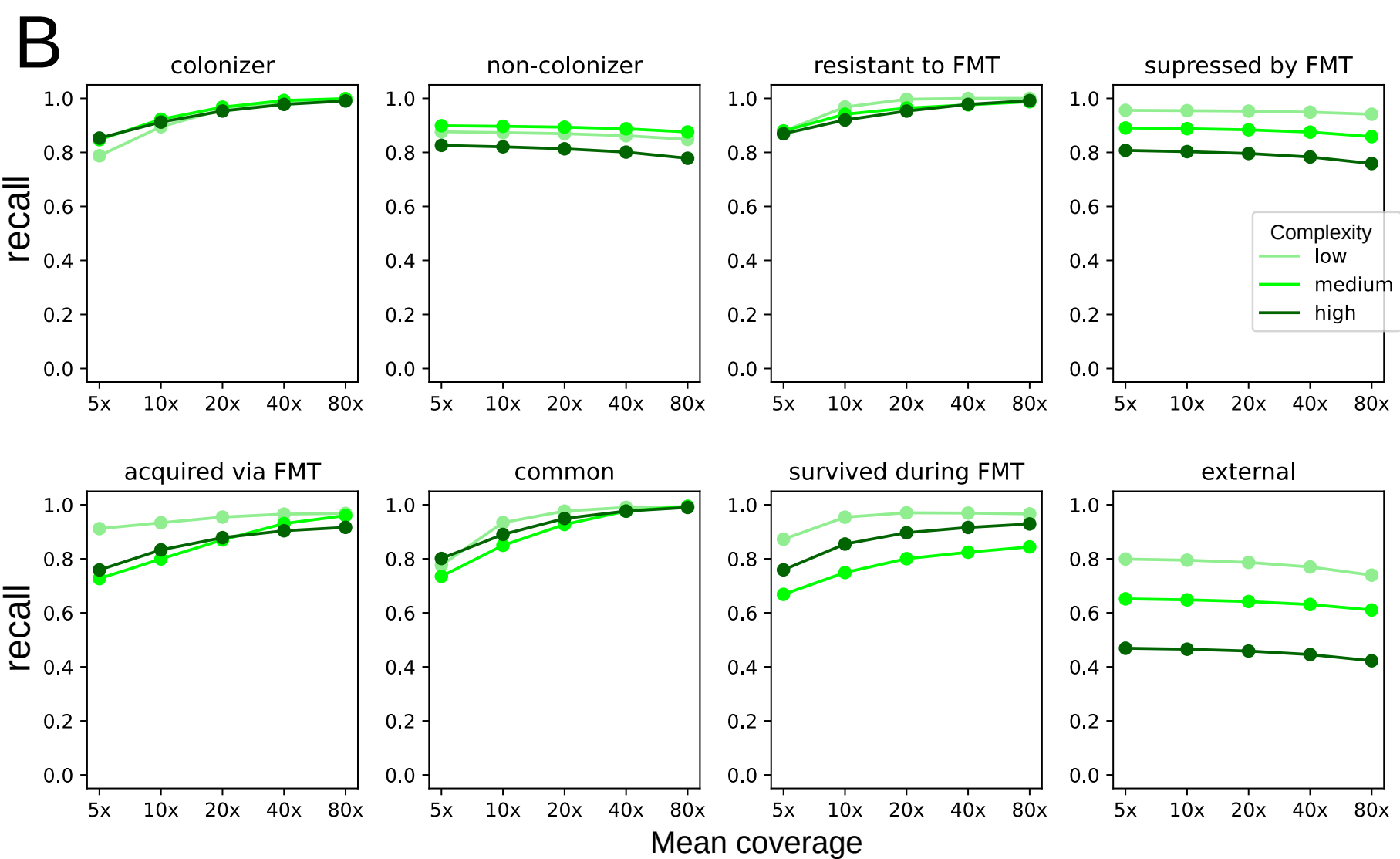

Supplement: FIG S2 [file msystems.00811-21-sf002.pdf]

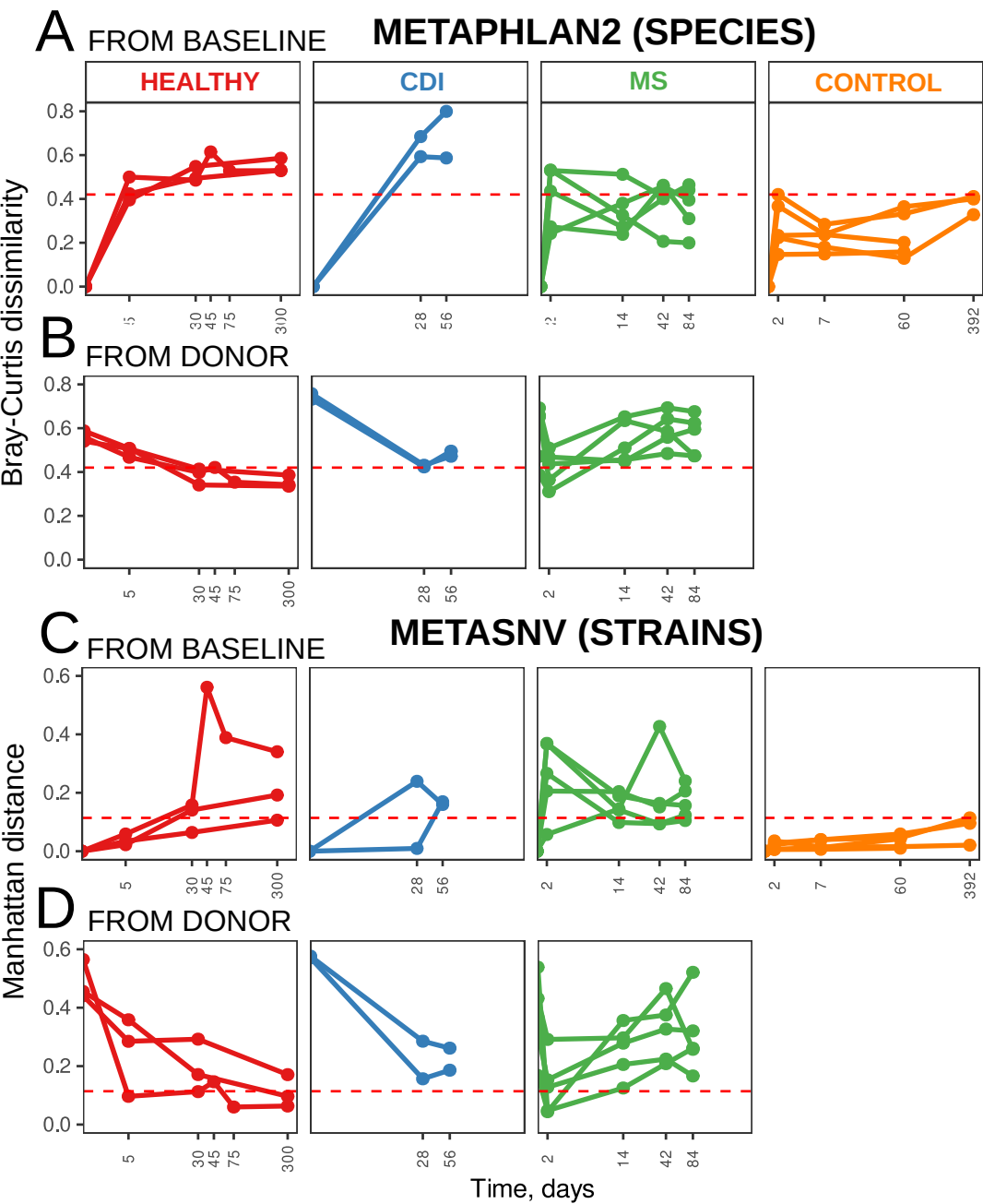

Supplement: FIG S3 [file msystems.00811-21-sf003.pdf]
